# Six1 and Six2 of the Sine Oculis Homeobox Subfamily are Not Functionally Interchangeable in Mouse Nephron Formation

**DOI:** 10.3389/fcell.2022.815249

**Published:** 2022-02-01

**Authors:** Jinshu Xu, Jun Li, Aarthi Ramakrishnan, Hanen Yan, Li Shen, Pin-Xian Xu

**Affiliations:** ^1^ Department of Genetics and Genomic Sciences, New York, NY, United States; ^2^ Department of Neurosciences, New York, NY, United States; ^3^ Department of Cell, Developmental and Regenerative Biology, Icahn School of Medicine at Mount Sinai, New York, NY, United States

**Keywords:** gene duplication, vertebrate evolution, Six1 and Six2, nephron progenitor, maintenance, differentiation

## Abstract

The vertebrate Six1 and Six2 arose by gene duplication from the *Drosophila sine oculis* and have since diverged in their developmental expression patterns. Both genes are expressed in nephron progenitors of human fetal kidneys, and mutations in SIX1 or SIX2 cause branchio-oto-renal syndrome or renal hypodysplasia respectively. Since ∼80% of SIX1 target sites are shared by SIX2, it is speculated that SIX1 and SIX2 may be functionally interchangeable by targeting common downstream genes. In contrast, in mouse kidneys, Six1 expression in the metanephric mesenchyme lineage overlaps with Six2 only transiently, while Six2 expression is maintained in the nephron progenitors throughout development. This non-overlapping expression between Six1 and Six2 in mouse nephron progenitors promoted us to examine if Six1 can replace Six2. Surprisingly, forced expression of Six1 failed to rescue Six2-deficient kidney phenotype. We found that Six1 mediated Eya1 nuclear translocation and inhibited premature epithelialization of the progenitors but failed to rescue the proliferation defects and cell death caused by Six2-knockout. Genome-wide binding analyses showed that Six1 selectively occupied a small subset of Six2 target sites, but many Six2-bound loci crucial to the renewal and differentiation of nephron progenitors lacked Six1 occupancy. Altogether, these data indicate that Six1 cannot substitute Six2 to drive nephrogenesis in mouse kidneys, thus demonstrating that the difference in physiological roles of Six1 and Six2 in kidney development stems from both transcriptional regulations of the genes and divergent biochemical properties of the proteins.

## Introduction

The duplication of developmental regulatory genes is one of the evolutionary driving forces leading to the diversity and complexity of higher eukaryotes. One of such multigene families encodes for the sine oculis (SIX) homeodomain transcription factors, which can be divided into three subfamilies—Six1/2, Six3/6, and Six4/5 based on their sequence and structure conservation (Kawakami et al., 2000; Kumar 2009). Each of these subfamilies is respectively duplicated from each of the three SIX genes in *Drosophila*—*so* (*sine oculis*), *ptix* (also known as DSix3), and DSix4 (Seo et al., 1999). These factors are found in diverse organisms ranging from flatworms to humans and are crucial for cell lineages that give rise to cranial sensory organs, brain, kidney, muscle and gonads. The three *Drosophila* SIX genes are thought to have arisen by duplication of a single ancestral gene, an event that occurred prior to the evolution of the Bilateria (Kawakami et al., 2000). Since the genome of most invertebrates contains only a single gene for each subfamily, the complexity of the SIX subfamilies has arisen by a further duplication of each SIX gene at the onset of vertebrate evolution.

The SIX family proteins are characterized by the presence of two evolutionarily conserved domains, the SIX-specific domain (SD) that mediates interactions with partner proteins and DNA binding homeodomain (HD). Both the N-terminus adjacent to the 5′ end of the SD and the C-terminus flanking the 3’ end of the HD are considerably variable in length with a very low degree of sequence conservation across all SIX family members (Kawakami et al., 2000; Kumar 2009). Evidence from multiple studies has revealed a remarkable similarity in the binding sites for Six1/2/4/5 (TCAGGTTC) (Suzuki-Yagawa et al., 1992; Kawakami et al., 1996; Spitz et al., 1998; Harris et al., 2000; Sato et al., 2002; Brodbeck et al., 2004). Analysis of the divergent C-terminal domains of the SIX proteins found that its presence increases the affinity of the HD for DNA (Hu et al., 2008). However, how the structural diversity of the C-terminal regions confers specificity in regulating target genes remains poorly understood.

Six1 and Six2 of the *so* subclass show spatiotemporally overlapping expression patterns in many tissues during development (Oliver et al., 1995; Abitua et al., 2015; Horie et al., 2018). For instance, SIX1 and SIX2 are coexpressed in human fetal nephron progenitors (O'Brien et al., 2016). Heterozygous SIX2 missense mutations were identified in patients with renal hypodysplasia characterized by reduced kidney size and/or maldevelopment of the renal tissue following abnormal organogenesis (Weber et al., 2008), while SIX1 mutations result in Branchio-Oto-Renal (BOR) or Branchio-Oto (BO) syndrome (Ruf et al., 2004; Okada et al., 2006; Kochhar et al., 2008), an autosomal dominant disorder characterized by abnormal development of the second branchial arch, otic with or without renal anomalies (Melnick et al., 1976; Fraser et al., 1980; Abdelhak et al., 1997a; Abdelhak et al., 1997b; Vincent et al., 1997; Kumar et al., 2000; Vervoort et al., 2002; Kochhar et al., 2007). Because of the overlapping expression of SIX1 and SIX2 in the fetal nephron progenitors, previous studies used chromatin immunoprecipitation followed by deep sequencing (ChIP-seq) to address how much diversity exists among their transcriptional targets and found that ∼80% of SIX1-bound sites are shared by SIX2 (O'Brien et al., 2016). This led to the speculation that SIX1 and SIX2 may be functionally interchangeable in targeting common downstream genes in the nephron progenitors, despite SIX2 binding to more sites. However, it is difficult to test this possibility and decipher the difference in their biochemical activities in the human kidney.

In contrast, the expression of Six1 and Six2 in the mouse kidney only transiently overlaps in the metanephric mesenchyme (MM) before the onset of ureteric bud (UB) branching (Li et al., 2003; Xu et al., 2003; Self et al., 2006; Nie et al., 2011; Park et al., 2012; O'Brien et al., 2016). Kidney organogenesis in mice commences when the MM induces the UB to outgrow from the nephric duct and invade into the MM at ∼ E10.5-E11.0. The UB tip cells then induce the MM to form the cap mesenchyme (CM, also called nephron progenitors) surrounding the UB tip and subsequent reciprocal interactions between the CM and the UB tip cells lead to repeated UB branching to form the nephron tubules and collecting duct system. Throughout development, high levels of the Six2 expression are maintained in the nephron progenitors (Self et al., 2006; Park et al., 2012; O'Brien et al., 2016). Consistent with these differential expression patterns, germline Six2 deletion in mice leads to renal hypoplasia due to depletion of the nephron progenitors (Self et al., 2006), while Six1 knockout causes renal agenesis due to malformation of the MM (Li et al., 2003; Xu et al., 2003; Kobayashi et al., 2007; Nie et al., 2011; Xu and Xu 2015).

The non-overlapping expression between Six1 and Six2 in mouse nephron progenitors promoted us to examine if the forced expression of Six1 in the nephron progenitors can rescue Six2-deficient kidney phenotype by crossing a Six1 knockin into Eya1 locus (Eya1^Six1^) mouse model (Nie et al., 2010) with *Six2* ^*+/−*^ mice (Self et al., 2006). Eya1 is coexpressed with Six2 in the MM progenitors throughout kidney development and it acts upstream of Six2, but their gene products interact and Six2 mediates Eya1 nuclear translocation (Xu et al., 1999; Xu et al., 2014). To our surprise, analysis of *Eya1* ^*Six1/+*^ *;Six2* ^*−/−*^ mice revealed that Six1 failed to rescue Six2-deficient kidney phenotype. We found that Six1 was able to mediate nuclear translocation of Eya1 and inhibit rapid premature differentiation of the progenitors that occurred in *Six2* ^*−/−*^ mice but failed to substitute for Six2 to renew and maintain the nephron progenitors. Hence, the Six1 and Six2 proteins are not functionally interchangeable in the nephron progenitors. We further performed ChIP-seq in ∼E13.5 Eya1^Six1/+^ kidneys to investigate if Six1 targets Six2-bound regulatory regions that are essential for the maintenance of nephron progenitors, as suggested in humans. Our analyses revealed that Six1 only selectively occupied a small subset of Six2 target sites, but many Six2-bound loci that are crucial to the renewal and differentiation of nephron progenitors lacked Six1 occupancy. Thus, these data demonstrate that Six1 and Six2 have not maintained equivalent biochemical properties since their divergence early in vertebrate evolution.

## Materials and Methods

### Animals and Genotyping

All animal protocols were approved by Animal Care and Use Committee of the Icahn School of Medicine at Mount Sinai (protocol #06-0822).

The Eya1^Six1/+^ (Nie et al., 2010) and *Six2* ^*+/−*^ (Self et al., 2006) were maintained at the Icahn School of Medicine at Mount Sinai Animal Facility. Mice were bred using timed mating, and noon on the day of vaginal plug detection was considered as E0.5.

### Histology, Immunohistochemistry, and *in situ* Hybridization

Histology, immunohistochemistry, and *in situ* hybridization were carried out according to standard procedures. Briefly, kidneys were fixed in 4% paraformaldehyde (PFA) for overnight at 4°C, dehydrated, and embedded in wax or OCT. Paraffin or frozen sections were generated at 6 μm of thickness. We used six embryos for each genotype at each stage for each probe and the result was consistent in each embryo. Probes for ISH were reported previously (Xu et al., 2014). Cy3-, Cy2-, Cy5-and FITC-conjugated secondary antibodies were used and Hoechst was used for nuclear counter-staining.

Primary antibodies: Anti-Six1 (12,891, Cell Signaling), -Six2 (MBS610128, MyBiosource), anti-Wt1 (sc-192, Santa Cruz Biotechnology), -Eya1 (25-067, Prosci Inc. and MABE1047, Sigma), and -PH3 (ab10543, Abcam).

### TUNEL Assays

The TUNEL assay was performed using the Apop Tag kit for *in situ* apoptosis fluorescein detection (S7110, Millipore-Sigma) following the manufacturer’s instructions.

### Cell Counts and Statistical Analysis

TUNEL- or PH3-positive MM progenitor cells on the peripheral side of branching UB were counted from 28 to 35 sections of 5 different kidneys and values represent average number of TUNEL^+^ or PH3^+^ cells (±standard deviations) per section (6 μm). Two-tailed Student’s *t* test was used for statistical analysis.

### Chromatin Immunoprecipitation Followed by Deep Sequencing

For Six1 ChIP, we used 40 kidneys from ∼E13.5 Eya1^Six1^ embryos. ChIP-seq was performed according to previous protocols with some modifications. Briefly, the kidneys were cross-linked with 1% formaldehyde at room temperature for 30 min and then homogenized and lysed in cold lysis buffer (50 mM HEPES–KOH, pH 7.5, 140 mM NaCl, 1 mM EDTA, 10% glycerol, 0.5% NP–40, 0.25% Triton X-100, 1 × protease inhibitors), followed by spinning at 2000 g at 4°C, resuspending in cold wash buffer (10 mM Tris–HCl, pH 8.0, 200 mM NaCl, 1 mM EDTA, 0.5 mM EGTA, 1 × protease inhibitors) and spinning at 2000 g at 4°C in a benchtop centrifuge. Samples were then resuspended in 1 ml cold sonication buffer (10 mM Tris–HCl, pH 8.0, 2 mM EDTA, 0.1% SDS) and sonicated to 200–500 bp fragments using a Covaris S220 Focused-ultrasonicator. Sonicated chromatin was cleared by pelleting insoluble material at 13,000 RPM at 4°C, followed by preclearing with protein A/G beads and incubation with 1–2 µg antibody overnight (anti-Six1, Cell Signaling #12891) or 1–2 µg rabbit IgG as a negative control. Chromatin–antibody complexes were precipitated with protein A/G beads at 4°C for another 5 h. Immunoprecipitated complexes were subjected to series of wash steps with low salt buffer (20 mM Tris–HCl 8.0, 150 mM NaCl, 2 mM EDTA, 1% Triton X-100, 0.1% SDS), high salt buffer (20 mM Tris–HCl pH 8.0, 500 mM NaCl, 2 mM EDTA, 1% Triton X-100, 0.1% SDS), LiCl wash buffer (10 mM Tris–HCl pH 8.0, 250mMLiCl, 1mMEDTA, 1%NP-40, 1% sodium deoxycholate) and TE plus NaCl, followed by elution and reverse crosslinking overnight at 65°C. The quality control of ChIPed DNA was performed with Qubit 2.0 Fluoremeter using dsDNA HS assay Kit (Q32854, ThermoFisher Scientific) and Agilent 2200 TapeStation System using High Sensitivity D1000 Reagents (5067–5585, Agilent). The pulldown and input control sequencing libraries were generated using the ThruPLEX DNA-seq Kit (R400429, Rubicon Genomics) and sequenced on Illumina NextSeq 500.

### Peak Calling, Annotation, and Motif Enrichment Analysis

Quality controls using FastQC (v0.11.2) (www.bioinformatics.babraham.ac.uk/projects/fastqc) were generated and raw sequencing reads were then aligned to the mouse mm10 genome using default settings of Bowtie (v2.2.0). Peak-calling was performed using MACS (v2.1.1) with various *p*-value cutoffs. The peak bed files were generated from peak calling against genomic input control or IgG control with the default setting (10^−5^ cutoff). The common peaks from these two bed files were used for subsequent analyses. The overlapping peaks of bed files were identified using the bedtools from Galaxy (https://usegalaxy.org/).

Motif enrichment analysis was performed using the Homer package (v4.8.3) (Heinz et al., 2010). The peak annotation and gene ontology analysis was performed using GREAT program (great.stanford.edu). The bamCoverage and coverage heatmap were generated by centering and scaling corrected ChIP-seq coverage using mean and standard deviation, and plotting normalized coverage across 3-kb centered on the Six1-enriched region.

### Data Access

ChIP-seq data sets have been submitted to the NCBI Gene Expression Omnibus (GSE189131).

## Results

### Forced Expression of Six1 in Nephron Progenitors Fails to Rescue *Six2* ^*−/−*^ Kidney Phenotype

The nephron progenitors on the peripheral side of the branching UB tip self-renew to replenish to generate a sufficient number of nephrons. In contrast, the progenitors on the ventral side of the branching UB tip undergo a mesenchyme-to-epithelial transition to form pretubular aggregates (PTAs), which then epithelialize to form renal vesicle (RVs)—the primordia of nephron tubules. Six2 is highly expressed in the multipotent nephron progenitors, and Six2 knockout leads to rapid RV formation and depletion of the nephron progenitors, resulting in hypoplastic kidneys (Self et al., 2006). To examine the functional equivalence between Six1 and Six2 during nephrogenesis, we tested whether forced expression of Six1 in the nephron progenitors can rescue Six2-deficient kidney phenotype by crossing *Six2* ^*+/−*^ mice with a Six1 knockin mouse model expressing Six1 under Eya1 transcriptional regulatory control (Eya1^Six1^) (Nie et al., 2010).

**FIGURE 1:**
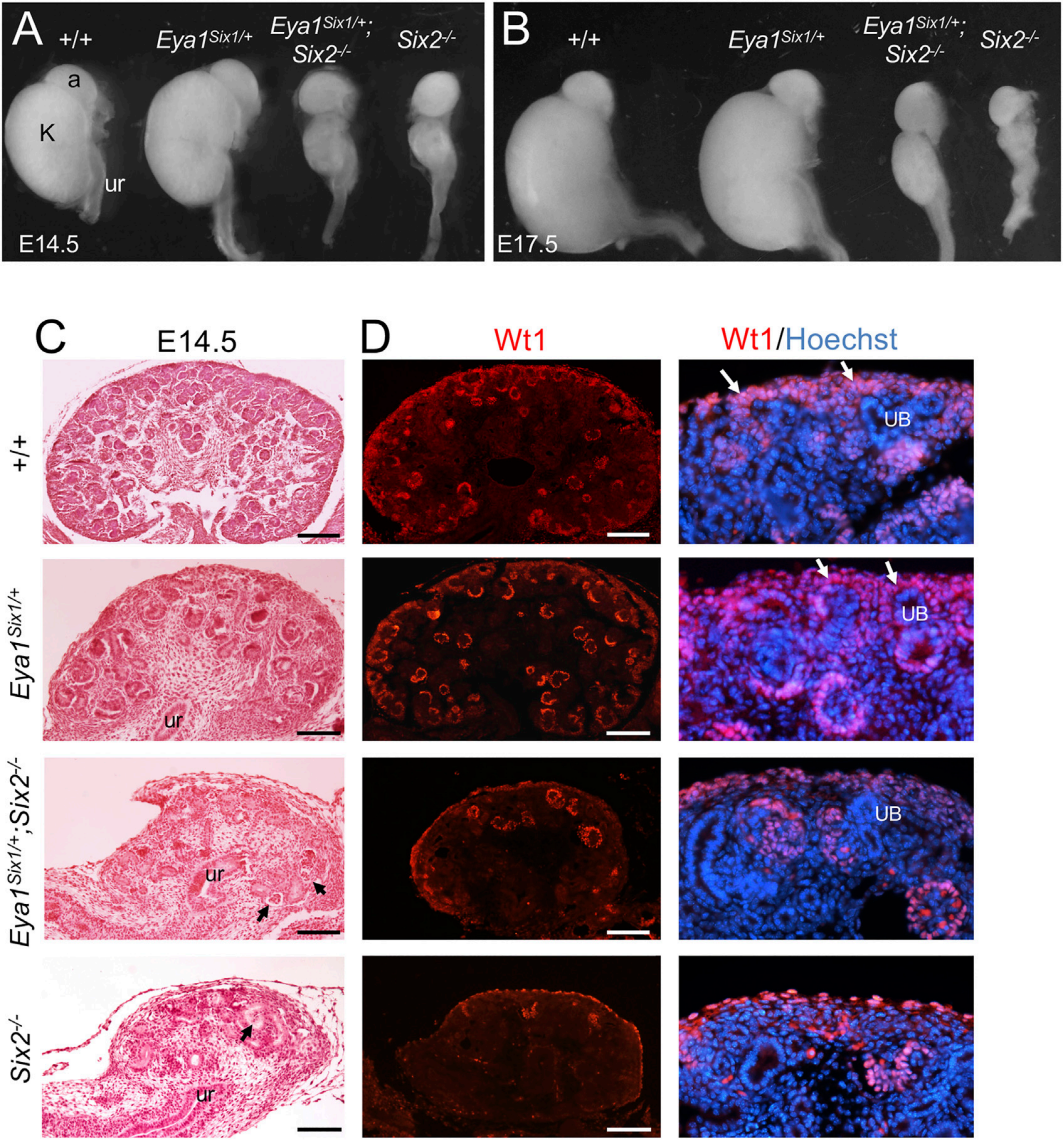
Forced expression of Six1 in the MM cells under Eya1 transcriptional control cannot rescue the Six2-deficient kidney phenotype. **(A,B)** Kidneys of wild-type, Eya1^Six1/+^, *Eya1* ^*Six1/+*^ *;Six2* ^*−/−*^ and *Six2* ^*−/−*^ at E14.5 and E17.5. **(C)** Hematoxylin and Eosin-stained sections of wild-type, Eya1^Six1/+^, *Eya1* ^*Six1/+*^ *;Six2* ^*−/−*^ and *Six2* ^*−/−*^ kidneys at E14.5. Arrows point to glomerulus-like structures. **(D)** Anti-Wt1 immunostaining on sections showing depletion of Wt1-labeled nephron progenitors (white arrows) in the outmost region of both *Eya1* ^*Six1/+*^ *;Six2* ^*−/−*^ and *Six2* ^*−/−*^ kidneys. Panels on the right are higher magnification of the panels on the left. Abb. a, adrenal gland; k, kidney; ub, ureteric bud; ur, ureter. Scale bars: 100 μm.

Eya1 is coexpressed with Six1 in the uninduced MM (Xu et al., 2003; Xu and Xu 2015) and with Six2 in the CM, and it is upstream of Six2 (Xu et al., 2014). We previously reported that renal agenesis associated with Six1-deficienc*y* can be rescued in *Eya1* ^*Six1/+*^ *;Six1* ^*−/−*^ mice (Nie et al., 2010). However, to our surprise, forced expression of Six1 failed to rescue Six2-deficient hypoplastic kidneys at E14.5–17.5 (*n* = 8, Figures 1A,B). Quantitative analysis of kidney size showed that the length of Eya1^Six1/+^ kidney was ∼104 ± 5% (*n* = 6, *p* = 0.0375) at E14.5 and ∼101 ± 3% (*n* = 8, *p* = 0.0418) at E17.5 of the wild-type, while the length of *Eya1* ^*Six1/+*^ *;Six2* ^*−/−*^ kidney was ∼106 ± 5% (*n* = 8, *p* = 0.0357) at E14.5 and ∼114 ± 7% (*n* = 8, *p* = 0.0208) of *Six2* ^*−/−*^ littermate. Histological analysis and immunostaining for Wt1, which is expressed in the CM, PTAs, RVs and differentiating podocytes, revealed that both *Eya1* ^*Six1/+*^ *;Six2* ^*−/−*^ and *Six2* ^*−/−*^ mice lacked the nephron progenitors in the outmost nephrogenic zone where the UB branching morphogenesis normally takes place (Figures 1C,D). Similar observation was obtained when we used Eya1^CreER^ to induce Six1 expression in the MM by tamoxifen administration (*Eya1* ^*CreER*^ *;R26-Six1mCherry*) and tested its ability to restore kidney development in *Six2* ^*−/−*^ (data not shown). Thus, these findings demonstrate that the expression of Six1 in the MM cells under the Eya1 transcriptional regulatory control cannot functionally substitute for Six2 to maintain the nephron progenitor cells during mouse kidney development.

### Six1 Inhibits Rapid Differentiation of the Nephron Progenitors Caused by Six2 Knockout

**FIGURE 2:**
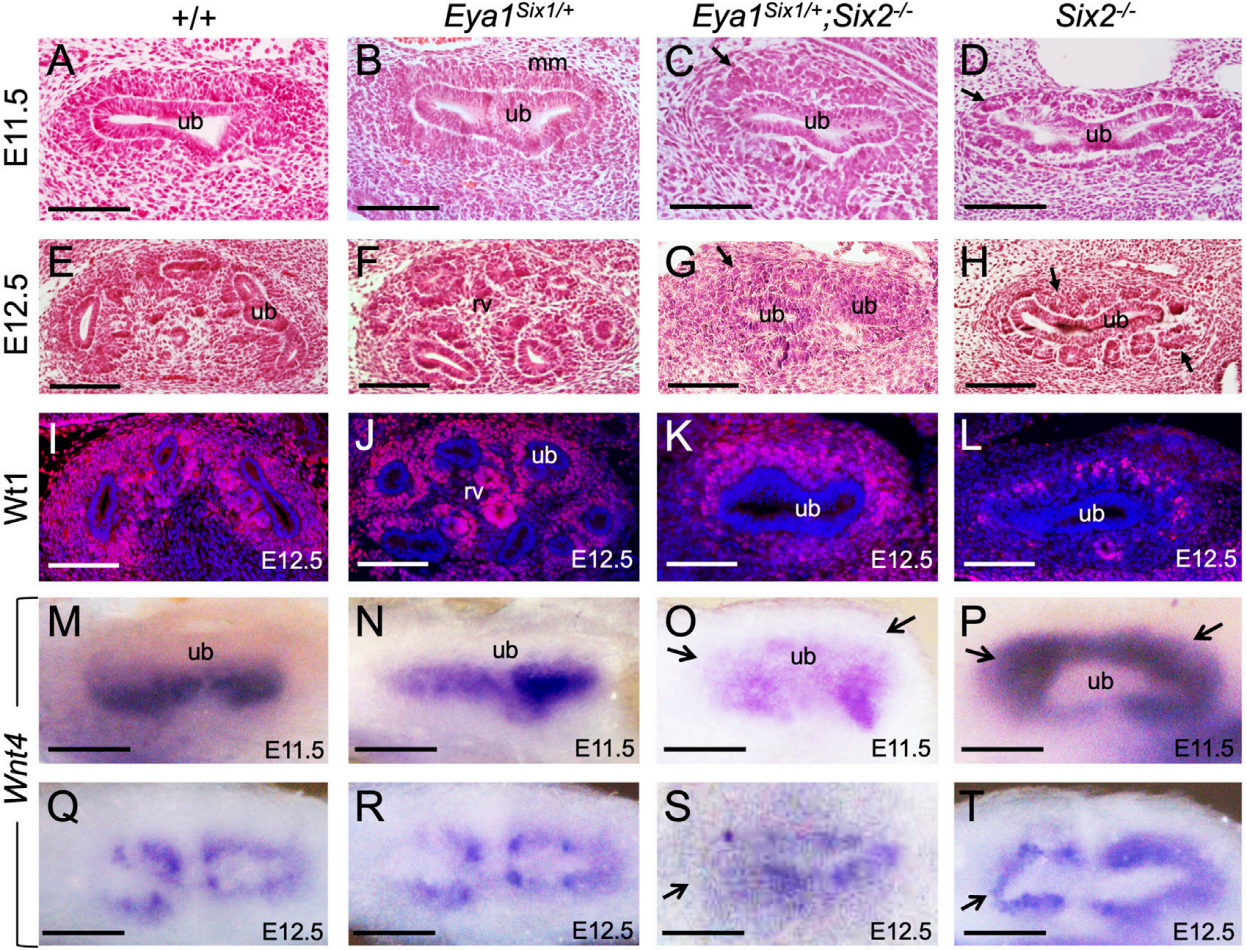
Forced expression of Six1 in the MM progenitors appears to inhibit their rapid epithelization induced by Six2 knockout. **(A–H)** Hematoxylin and Eosin-stained kidney sections of wild-type **(A,E)**, Eya1^Six1/+^ **(B,F)**, *Eya1* ^*Six1/+*^ *;Six2* ^*−/−*^ **(C,G)** and *Six2* ^*−/−*^ **(D,H)** at E11.5 and E12.5. *Six2* ^*−/−*^ displayed aggregates at E11.5 and RVs at E12.5 in the MM (metanephric mesenchyme) surrounding the branching UB (ureteric bud) (arrows in D,H). Arrows in panels C and G indicate the absence of PTAs or RVs within the MM cells of *Eya1* ^*Six1/+*^ *;Six2* ^*−/−*^. **(I–L)** Anti-Wt1 immunostaining on sections of wild-type, Eya1^Six1/+^, *Eya1* ^*Six1/+*^ *;Six2* ^*−/−*^, and *Six2* ^*−/−*^ kidneys at E12.5. Note that the kidney development in panel K was slightly delayed. **(M–T)** Whole-mount *in situ* hybridization showing Wnt4 expression in differentiating progenitors in wild-type, Eya1^Six1/+^ and *Eya1* ^*Six1/+*^ *;Six2* ^*−/−*^ and its global activation in *Six2* ^*−/−*^ MM at E11.5 and E12.5. Arrows in O and S indicate the absence of Wnt4 activation in the MM progenitors at UB tips. Arrows in P and T indicate ectopic Wnt4 activation in the MM of *Six2* ^*−/−*^ embryos. Scale bars: 100 μm.

We next examined whether there is a partial rescue at earlier stages, from the first “T” bud stage at E11.5. As reported previously (Self et al., 2006), the progenitors within the entire MM surrounding the branching UB underwent premature differentiation to form PTA- or RV-like structures in *Six2* ^*−/−*^ embryos at E11.5 (Figure 2D) and RV-like structures surrounded the entire UB at E12.5 (Figures 2H,L). In contrast, *Eya1* ^*Six1/+*^ *;Six2* ^*−/−*^ MM progenitors surrounding the branching UB appeared less dense than those in Eya1^Six1/+^ or wild-type littermate controls at E11.5 (Figures 2A–C), but they did not commit to an overall RV fate, as shown by histological analysis and anti-Wt1 immunostaining (Figures 2C,G,K). This observation was consistent with the expression pattern of Wnt4, which is one of the earliest markers labeling differentiating nephron structure PTAs at E11.5 and RVs at E12.5 on the ventral side of the branching UB (Figures 2M,N,Q,R). Differing from the relatively uniform expression of Wnt4 throughout the entire MM in *Six2* ^*−/−*^ embryos (Figures 2P,T), *Eya1* ^*Six1/+*^ *;Six2* ^*−/−*^ embryos did not display a global activation of Wnt4 in the entire MM and higher levels of Wnt4 expression was detected on the ventral side of the branching UB at E11.5 (Figure 2O). However, the pattern of Wnt4 expression appeared abnormal at E12.5 (Figure 2S) compared to those in Eya1^Six1/+^ littermate controls (Figures 2Q,R). By E12.5, the UB typically has completed second branching in control embryos (Figures 2E,F,I,J). In *Six2* ^*−/−*^ embryos, the first branching T-shaped UB appeared as a single tube, and the UB tips were not induced to undergo second branching due to the depletion of the MM progenitors (Figures 2D,H,L,P,T). In contrast, *Eya1* ^*Six1/+*^ *;Six2* ^*−/−*^ UB tips appeared to be induced and expanded to invade the MM to initiate (Figure 2K) or undergo second branching (Figure 2G), but the second branching was either incomplete or abnormal. Thus, while the UB development in *Eya1* ^*Six1/+*^ *;Six2* ^*−/−*^ is arrested at the second branching, Six1 expression in *Six2* ^*−/−*^ MM appears to prevent premature mesenchyme-to-Epithelial Transition

### Six1 Cannot Substitute for Six2 to Maintain the Nephron Progenitors

**FIGURE 3:**
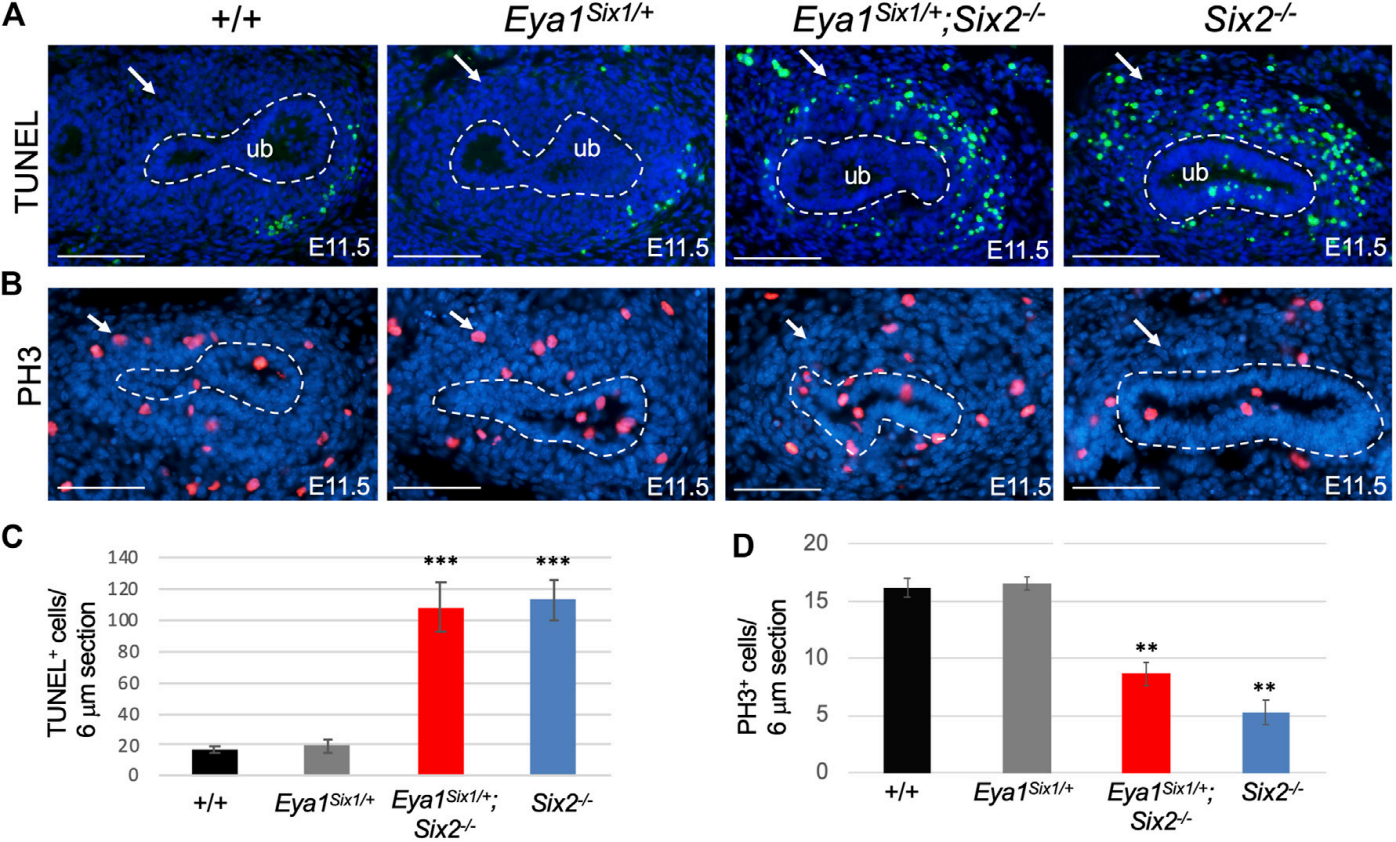
Six1 fails to rescue defective progenitor cell proliferation and cell death associated with Six2-deficiency. **(A,C)** TUNEL labeling on kidney sections at E11.5 showing increased apoptosis in the MM of *Eya1* ^*Six1/+*^ *;Six2* ^*−/−*^ and *Six2* ^*−/−*^ mutants (arrows in A), and quantification of TUNEL^+^ cells in the MM **(C)**. Note that increased apoptosis was also observed in the UB of *Six2* ^*−/−*^ embryos. **(B,D)** Anti-PH3 (Phosphohistone H3) staining showing reduced PH3^+^ mitotic cells in the MM of *Eya1* ^*Six1/+*^ *;Six2* ^*−/−*^ and more reduced in *Six2* ^*−/−*^ mutant than in wild-type or Eya1^Six1/+^ controls (arrows in B) and quantification of PH3^+^ mitotic MM cells **(D)**. Scale bars: 100 μm. For quantification, TUNEL^+^ or PH3^+^ MM progenitor cells were counted from 28 to 35 sections of 5 different kidneys and values represent average number of TUNEL^+^ or PH3^+^ cells (±standard deviations) per section (6 μm). Data from Eya1^Six1/+^, *Eya1* ^*Six1/+*^ *;Six2* ^*−/−*^ or *Six2* ^*−/−*^ were compared with wild-type. ***p-value < 0.001 and **p-value < 0.01.

As we noticed that the MM in *Eya1* ^*Six1/+*^ *;Six2* ^*−/−*^ is not as dense as in Eya1^Six1/+^, we sought to examine whether Six1 can rescue cell survival or proliferation defects in *Six2* ^*−/−*^ embryos. TUNEL (terminal deoxynucleotidyl transferase dUTP nick end labeling) assay revealed a noticeable degree of apoptosis throughout the MM areas, as a marked increase in the number of TUNEL^+^ cells in the MM was detected in both *Eya1* ^*Six1/+*^ *;Six2* ^*−/−*^ and *Six2* ^*−/−*^ (Figures 3A,C). It should be noted that increased apoptosis was also observed in the branching UB of *Six2* ^*−/−*^ embryos at E11.5 (Figure 3A). In contrast, anti-PH3 (phosphohistone H3)-labeled mitotic cells in the MM were decreased in *Eya1* ^*Six1/+*^ *;Six2* ^*−/−*^ and more decreased in *Six2* ^*−/−*^ littermate embryos (Figures 3B,D). These data suggest that while Six1 appears to prevent premature epithelialization of the progenitors, it is not able to substitute the essential role of Six2 in nephron progenitor renewal and survival, thus leading to depletion of the nephron progenitors and arrest of nephrogenesis during second UB branching in *Eya1* ^*Six1/+*^ *;Six2* ^*−/−*^ embryos.

### Six1 Mediates Nuclear Localization of Eya1 in the Nephron Progenitors

**FIGURE 4:**
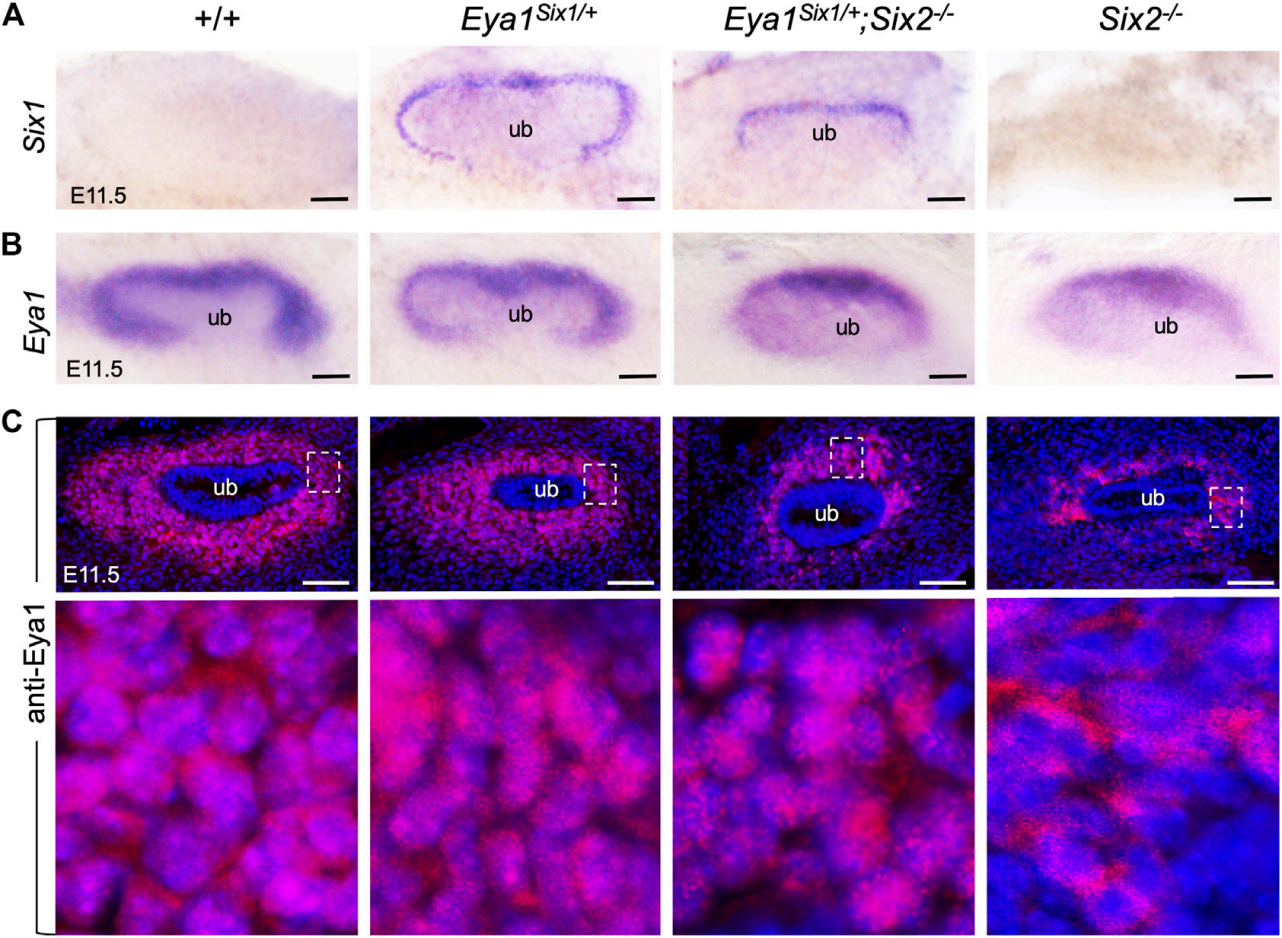
Six1 can mediate the nuclear translocation of Eya1 in the absence of Six2. **(A,B)** Whole-mount *in situ* hybridization for Six1 **(A)** and Eya1 **(B)** in the kidney rudiments of wild-type, *Eya1* ^*Six1/+*^ *, Eya1* ^*Six1/+*^ *;Six2* ^*−/−*^ and *Six2* ^*−/−*^ embryos at E11.5. **(C)** Anti-Eya1 immunostaining on kidney sections of E11.5 wild-type, *Eya1* ^*Six1/+*^ *, Eya1* ^*Six1/+*^ *;Six2* ^*−/−*^ and *Six2* ^*−/−*^ embryos. Lower panels are higher magnification of the boxed areas. Scale bars: 50 μm.

Loss of either Eya1 or Six2 in the nephron progenitors leads to increased cell death and reduced proliferation (Figure 3) (Self et al., 2006; Xu et al., 2014). We therefore performed ISH to confirm Six1 expression in the MM progenitors on the peripheral side of the branching UB in Eya1^Six1/+^ at E11.5, compared to no expression in wild-type or *Six2* ^*−/−*^ littermates (Figure 4A). In *Eya1* ^*Six1/+*^ *;Six2* ^*−/−*^, the MM appeared smaller in size as outlined by the Six1 expression (Figure 4A). We previously reported that although Eya1 mRNA is expressed in *Six2* ^*−/−*^ MM progenitors, Eya1 protein is localized in the cytoplasm of *Six2* ^*−/−*^ MM progenitors and its nuclear translocation depends on Six2 activity (Xu et al., 2014). In *Eya1* ^*Six1/+*^ *;Six2* ^*−/−*^ embryos, the Eya1^+^ domain was comparable to that seen in *Six2* ^*−/−*^ littermates, both of which were smaller in size than in control littermates (Figure 4B). Notably, however, anti-Eya1 immunostaining revealed nuclear localization of Eya1 in *Eya1* ^*Six1/+*^ *;Six2* ^*−/−*^ MM progenitors, in contrast to its cytoplasmic localization in *Six2* ^*−/−*^ MM cells (Figure 4C). Thus, Six1 is also able to mediate nuclear translocation of Eya1 in the progenitors in the absence of Six2. This observation is consistent with the previous finding that Six1 or Six2 but not Six3 can mediate nuclear translocation of Eya1 when coexpressed in HEK293 cells (Buller et al., 2001).

**FIGURE 5:**
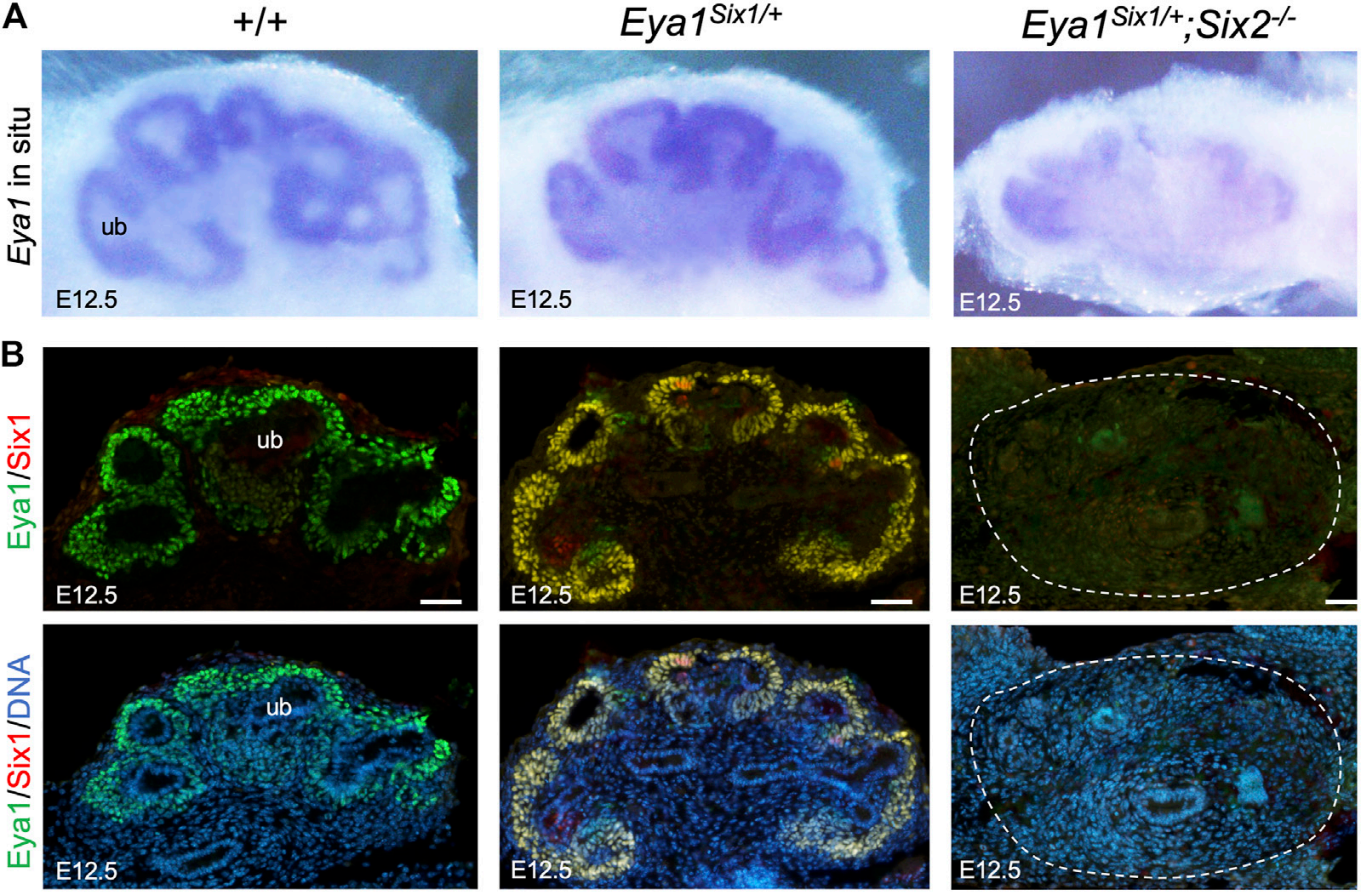
Eya1 protein expression is not maintained at later stages in the absence of Six2. **(A)** Whole-mount *in situ* hybridization for Eya1 in the kidneys of wild-type, Eya1^Six1/+^, and *Eya1* ^*Six1/+*^ *;Six2* ^*−/−*^ at E12.5. **(B)** Anti-Eya1 and -Six1 co-immunostaining on kidney sections of E12.5 wild-type, Eya1^Six1/+^, and *Eya1* ^*Six1/+*^ *;Six2* ^*−/−*^ embryos. Hoechst was used for nuclear counter-staining. ub, ureterci bud. Scale bars: 50 μm.

We further examined whether Eya1 expression can be maintained in the MM progenitors of *Eya1* ^*Six1/+*^ *;Six2* ^*−/−*^ embryos at later stages. As shown by whole-mount ISH (Figure 5A), Eya1 expression was strongly detected in the MM progenitors of wild-type or Eya1^Six1/+^ littermate kidneys at E12.5, but only residual Eya1 transcripts were observed in *Eya1* ^*Six1/+*^ *;Six2* ^*−/−*^ kidneys. Consistent with our previous observation (Xu et al., 2014), anti-Eya1 immunostaining revealed higher levels of Eya1 protein expression in the CM and lower levels in the differentiating RVs of wild-type or Eya1^Six1/+^ kidneys (Figure 5B). Co-immunostaining with a Six1-specific antibody confirmed co-expression of Six1 with Eya1 in Eya1^Six1/+^ but not in wild-type kidneys (Figure 5B). However, in *Eya1* ^*Six1/+*^ *;Six2* ^*−/−*^ kidneys, neither Eya1 nor Six1 protein expression was observed. Thus, Six1 cannot replace Six2 to maintain Eya1 expression in the MM progenitors in *Eya1* ^*Six1/+*^ *;Six2* ^*−/−*^ at E12.5.

Genome-wide ChIP-seq analysis identifies selective binding of Six1 to a subset of Six2 targets essential for nephron progenitor cell maintenance but no co-occupancy of Six1 with Six2 to genes involved in nephron differentiation such as Wnt4, Fgf8, and Pax8.

**FIGURE 6:**
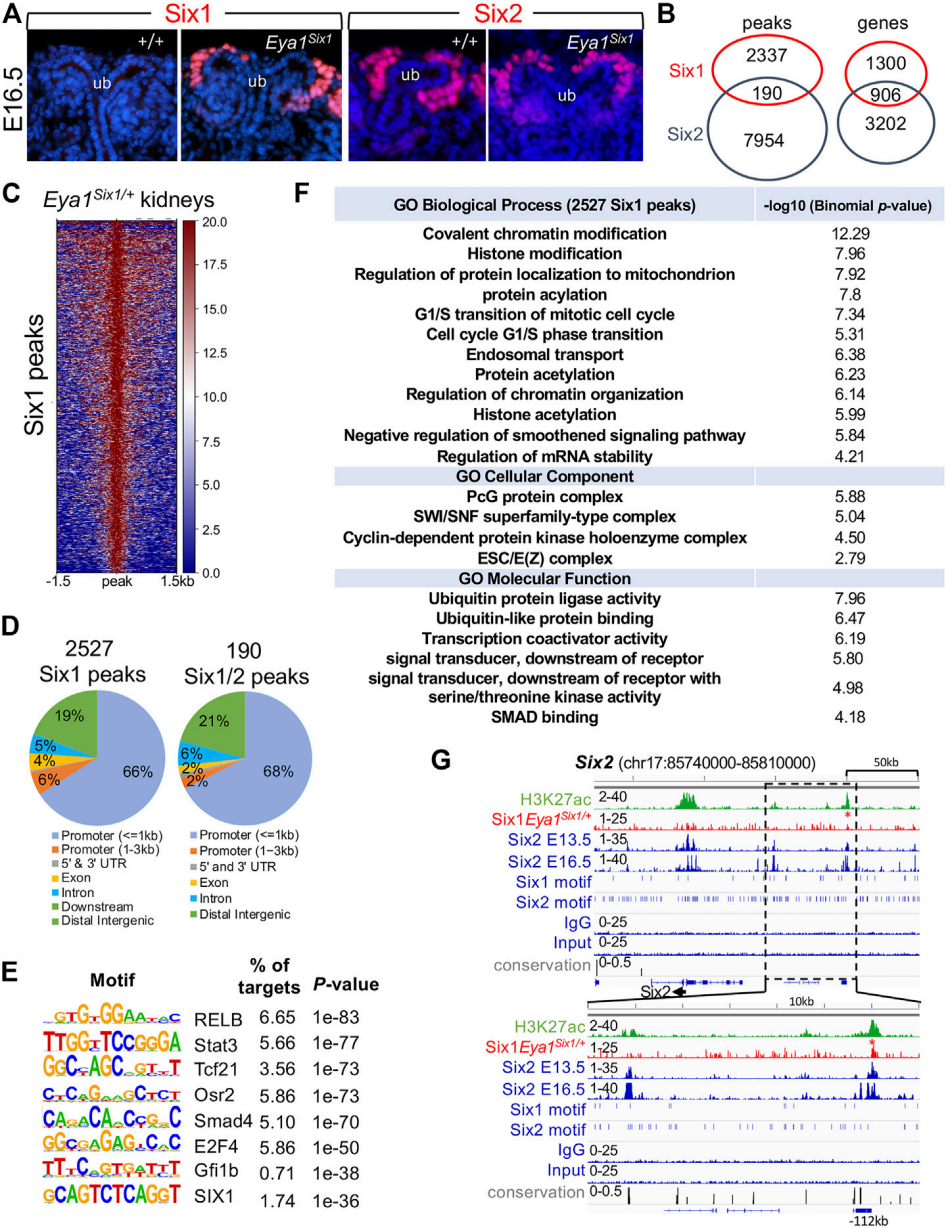
Anti-Six1 immunostaining reveals the stability of overexpressed Six1 protein in the nephron progenitors, and genome-wide Six1 binding analysis shows occupancy by Six1 in E13.5 Eya1^Six1/+^ kidneys. **(A)** Immunostaining with a Six1-or Six2-specific antibody showing high levels of the overexpressed Six1 in the cap mesenchyme (CM) surrounding the tips of the ureteric bud (UB) of Eya1^Six1/+^ kidneys at E16.5, similar to that of Six2 expression. **(B)** Venn diagram indicating overlap of Six1-and Six2-binding sites or putative targeted genes in E13.5 kidneys. **(C)** Heatmaps showing Six1 peaks within a -1.5-kb/+1.5-kb window centered on all peaks in Eya1^Six1/+^ kidneys. **(D)** Genomic distribution of Six1 or common Six1/Six2 peaks. UTR, untranslated region. **(E)** Sequence logos of the top enriched motifs identified from Homer *de novo* motif analysis, letter size indicates nucleotide frequency. Percentage of target sites in Six1 peaks with the significance of motif occurrence (*p*-value) is indicated. **(F)** Gene ontology (GO) analysis of Six1 peaks. **(G)** Genomic browser visualization of weak Six1 enrichment at a distal region ∼112-kb upstream of the Six2 promoter (red asterisk). This region was co-occupied by Six2 in E13.5 or E16.5 kidneys and associated with histone mark H3K27ac. The direction of transcription is shown by the arrow beginning at the TSS. The lower graph indicates a higher magnification of the boxed area. H3K27ac and Six2 ChIP-seq data (GSE185050) and Six2E16.5 (GSE73867) were used for comparison.

Our data suggest that Six1 cannot drive developmental programs regulated by Six2 to expand and maintain the nephron progenitors, thus implying that Six1 cannot target Six2-occupied sites in the nephron progenitors. In the human kidney, while ChIP-seq identified more SIX2 binding sites than SIX1, ∼81% (1307 of 1610 peaks) of SIX1 peaks overlapped with SIX2 peaks (∼20.8% of 6276 SIX2 peaks) (O'Brien et al., 2016). However, the functional cooperation and relative contributions of SIX1 versus SIX2 to progenitor cell maintenance and differentiation remain unknown. To directly address the potential functional differences between the mouse Six1 and Six2, we performed ChIP-seq to map Six1-bound regions genome-wide in Eya1^Six1/+^ kidneys. Immunohistochemistry with a Six1-specific antibody confirmed Six1 protein expression in the CM of Eya1^Six1/+^ at E16.5 (Figure 6A), indicating the stability of the overexpressed Six1 protein in the nephron progenitors. We then used kidneys ∼ E13.5 for Six1 ChIP-seq to avoid differentiating structures, and the peak bed files were generated from MACS peak calling against both genomic input DNA and IgG ChIP-seq controls with the default setting (10^−5^ cutoff). Peak calling identified 148 and 2527 Six1 peaks respectively from two different datasets, and we focused on the 2527 peaks (2206 genes) for subsequent analyses and comparison with our recent Six2 ChIP-seq data sets on wild-type kidneys at the same developmental stage (Li et al., 2021) (Figures 6B,C, Supplementary Tables S1,S2). Only 190 Six1 regions (∼7.5%) overlapped with Six2 peaks (Supplementary Table S3), but ∼41% of putative Six1 target genes (906 out of 2206 genes) were shared with Six2 (Supplementary Table S4). While a significant fraction of Six1 peaks were located near the promoter, ∼29% were found between 5–500 kb of TSSs (transcriptional start sites) and the majority of these peaks were distributed within intronic and intergenic regions (Figure 6D). Homer *de novo* motif search revealed that Six1 or Six2 motifs were not among the top mostly enriched motifs in the Six1 peaks (Figure 6E). Motifs for transcription regulators RELB, Stat3, Tcf21, Osr2, and Smad4 are among the top five enriched motifs. Gene Ontology (GO) analyses using GREAT did not identify nephron-specific terms, but revealed overrepresentation of genes associated with chromatin modification, histone modification, and transition of mitotic cell cycle (Figure 6F).

**FIGURE 7:**
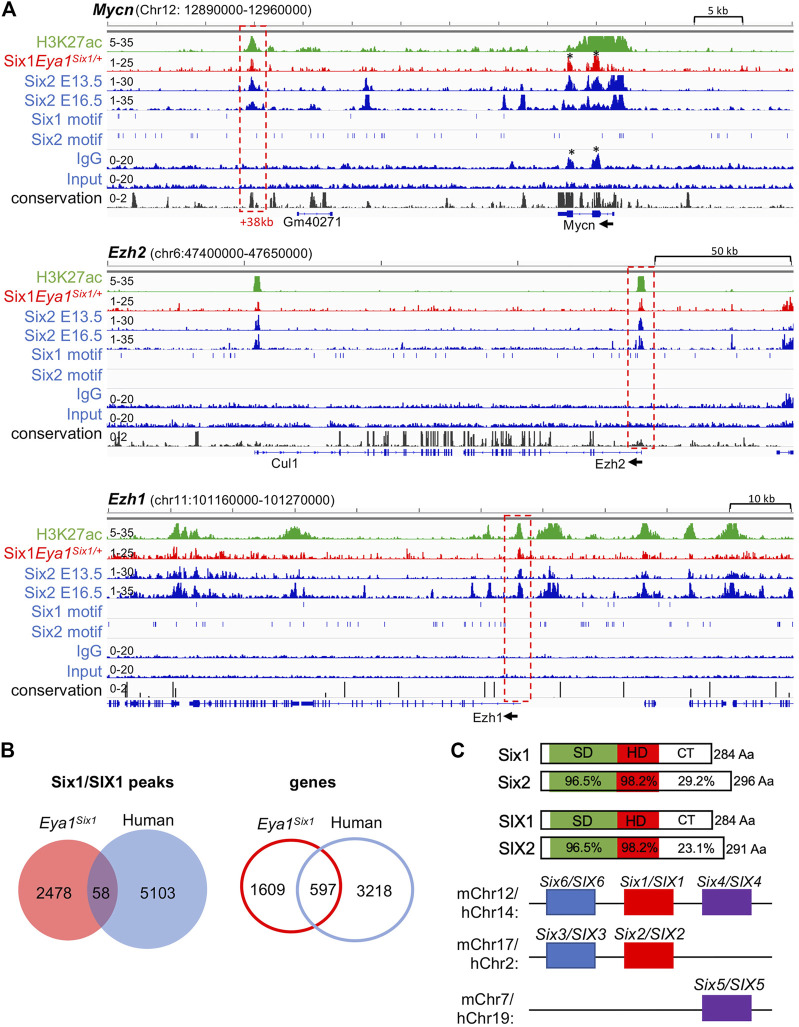
Six1 binds to Six2 targeted sites associated with H3K27ac-deposition at Mycn, Ezh1, and Ezh2. **(A)** Genome browser visualization of overlapping occupancy of Six1 and Six2 to a conserved enhancer region ∼38-kb downstream of the Mycn promoter and the proximal-promoter region of Ezh2 or Ezh1 (boxed by red dashed line). Black asterisks indicate non-specific enrichment peaks, as also seen in the IgG control. The direction of transcription is shown by the arrow beginning at the TSS. **(B)** Venn diagram indicating overlap of mouse Six1-and human fetal kidney SIX1-binding sites or targeted genes. **(C)** Schematic diagrams of mouse Six1/Six2 and human SIX1/SIX2 proteins and mouse or human chromosome locations of all three SIX subfamilies. SIX-specific domain (SD) and homeodomain (HD) are indicated. Note that the C-terminal regions (CT) are divergent in length with a low sequence conservation.

Eya1 is a critical factor for the maintenance of the nephron progenitors and interacts with Myc and Six2 (Xu et al., 2014), but Six2 is likely involved in maintaining high levels of Eya1 expression as Six2 binding to multiple regions at the Eya1 locus were identified in E13.5 and E16.5 kidneys (Park et al., 2012; O'Brien et al., 2016; Li et al., 2021). Our data suggest that Six1 cannot replace Six2 to maintain Eya1 expression in the progenitors (Figure 5), we therefore asked if Six1 can target the Six2-bound regions at the Eya1 locus to replace the Six2 function in enhancing Eya1 regulatory expression. However, peak calling did not identify Six1 occupancy to the Eya1 locus (Supplementary Table S2). Among the 190 regions shared between Six1 and Six2, Six1 enrichment was identified at ∼112-kb upstream of the Six2 promoter (Figure 6G), representing one of the several Six2 targeted sites with the active histone mark H3K27ac deposition. Similarly, Six1 enrichment was identified at a distal region ∼38-kb downstream of the Mycn promotor (Figure 7A), which is one of the several Six2-bound sites at the Mycn locus and is a highly conserved region associated with H3K27ac-deposition. Thus, Six1 appears to selectively target a small subset of Six2-bound sites at genes essential for nephron progenitor maintenance, which may explain why the number of mitotic MM progenitor cells was more reduced in *Six2* ^*−/−*^ than in *Eya1* ^*Six1/+*^ *;Six2* ^*−/−*^ (Figure 3D). The lack of Six1 co-occupancy at Six2-target sites of the Eya1 locus may at least partially lead to the disappearance of *Eya1* expression in *Eya1* ^*Six1/+*^ *;Six2* ^*−/−*^.

Previous studies have suggested a role for Six2 in inducing nephron differentiation as Six2 targets regions associated with H3K27ac-deposition at genes essential for nephron differentiation, such as Fgf8, Pax8, and Wnt4 (Park et al., 2012; O'Brien et al., 2016). In contrast, Six1 binding to these putative Six2 target genes was not observed (Supplementary Table S2). Interestingly, however, co-occupancy of Six1 with Six2 was identified at the promotor regions of the enhancer of zeste homolog Ezh1 and Ezh2, which had H3K27ac-deposition (Figure 7A). Ezh1 and Ezh2 are polycomb histone methyltransferases, and both are required for the renewal potential of nephron progenitors and co-regulate chromatin accessibility (Liu et al., 2020). Since inactivation of both Ezh1 and Ezh2 triggers unscheduled activation of Wnt4-driven differentiation and results in early termination of nephrogenesis (Liu et al., 2020), Six1 binding to these two genes could maintain the expression of both Ezh1 and Ezh2 in the absence of Six2, which in turn prevents ectopic activation of Wnt4 in the nephron progenitors in *Eya1* ^*Six1/+*^ *;Six2* ^*−/−*^ kidneys.

We further compared with human SIX1 ChIP-seq data in the fetal kidneys and found only 58 (∼2.3%) of mouse Six1 peaks overlapped with human SIX1-binding sites and ∼27% of putative Six1 target genes shared with human SIX1 (Figure 7B, Supplementary Table S5,S6), despite the high conservation between Six1 and SIX1 (Figure 7C). Previous studies identified only ∼8% of mouse Six2-binding sites but ∼50% of putative Six2 target genes overlapped with human SIX2 targets (O'Brien et al., 2016). Sequence comparison between these proteins revealed that the C-terminal domain is quite divergent in length between Six1 and Six2 with a low degree of sequence similarity, and the C-terminus of human SIX2 is shorter than the mouse Six2 (Figure 7C). This structural difference may affect the stability of forming complexes with specific partner proteins, thus resulting in Six1 selectively binding to only a small subset of Six2 target sites.

## Discussion

Six1 and Six2 genes are expressed in the MM progenitors during mouse kidney development, but Six1 is expressed before Six2 transcription and overlaps with Six2 only transiently before the onset of UB branching. *Six1* ^*−/−*^ embryos fail to form a functional MM competent for inducing UB branching, whereas *Six2* ^*−/−*^ mice exhibit later defects associated with expansion and maintenance of the nephron progenitors. Previous studies found that Six1 also cooperates with Six4 to regulate the formation of the MM as this structure is not formed in *Six1* ^*−/−*^ *;Six4* ^*−/−*^ mice (Kobayashi et al., 2007; Xu and Xu 2015), while *Six4* ^*−/−*^ mice are viable and normal (Ozaki et al., 2001). In this study, we overexpressed Six1 in the MM progenitors under Eya1 regulatory transcriptional control and examined the functional interchangeability between Six1 and Six2. Our data show that Six1 cannot fully rescue the Six2-deficient kidney phenotype, thus indicating that the different physiological roles of Six1 and Six2 relate to differences in both transcriptional regulations of the genes and divergent biochemical properties of the proteins.

From the sequence alignment of Six1 and Six2, it is evident that these two proteins share the highly conserved SD and HD regions (Figure 7C), except for the C-terminal domains that differ in length with a low degree of similarity. Since the HDs have very low intrinsic sequence specificity (Treisman et al., 1992), such structural differences adjacent to the HDs are likely to modify the specificity of the proteins by influencing the stability of complexes formed with particular partner proteins, thus affecting the ability of Six1 to compensate for Six2 loss. Based on our data, Six2 appears to have distinct and non-interchangeable roles in the multipotent nephron progenitors. Although Six1 can mediate nuclear translocation of Eya1 in the absence of Six2 (Figure 4), it apparently cannot act as a substitute for Six2 in maintaining Eya1 expression and driving the expansion and survival of the progenitors. This could be due to distinct stabilities of transcriptional complexes formed with partner proteins rather than differences in DNA-binding site preference, which explains why Six1 only selectively targets a small subset of Six2-bound regions and why Six1 cannot bind to Six2-target sites at the loci of Eya1 and Mycn. Consistent with this view, our previous studies testing the efficiency of coexpression of different members of the Eya family with various members of the SIX protein family in inducing hair cell development found that the combination of Eya1/Six1 acts most efficiently to activate a specific downstream regulatory program controlling inner ear hair cell or spiral neuron induction in a cochlear explant system (Ahmed et al., 2012a; Ahmed et al., 2012b). We found that ∼90% of Eya1/Six1-cotransfected cochlear nonsensory epithelial cells became Myo7a^+^ hair cells and that this function of Eya1 or Six1 is non-interchangeable with Eya2 or Six2, as only ∼3% of Eya1/Six2-or ∼6% of Eya2/Six1-cotransfected cochlear nonsensory epithelial cells became Myo7a^+^ hair cells (Ahmed et al., 2012a). Thus, we speculate that the divergent C-terminus of Six1 and Six2 may confer differential specificity in transcriptional complex formation and subsequent DNA recognition. Six1 may fail to collaborate with Six2-interacting transcription factors or chromatin regulators to generate a permissive chromatin context in the multipotent progenitors necessary for their renewal and survival. Although we do not understand why the mouse Six1 does not bind to many Six2 target sites that are co-occupied by human SIX1/SIX2, the simplest explanation is that there may be species-specific factors that affect the ability of Six1/SIX1 to target Six2/SIX2 sites. This may also explain why only ∼8% of mouse Six2-binding sites overlapped with human SIX2 peaks (O'Brien et al., 2016). Interestingly, while conserved, the C-terminus of Six2 also differs in length between the two species (Figure 7C). Human SIX2 is five amino acid residues shorter than mouse Six2, shortening the length difference between human SIX1 and SIX2. Thus, a comparative study with different SIX family proteins in transactivating nephron progenitor-specific target genes may give useful information about the importance of the C-terminal region.

Previous studies indicated that Six2 is likely to induce nephron commitment and differentiation by regulating the expression of target genes involved in nephron differentiation, such as Fgf8, Pax8, and Wnt4 (Park et al., 2012). Although *Six2* ^*−/−*^ nephron progenitors undergo premature differentiation, it is still unclear how Six2 acts to counter nephron differentiation. In contrast, Six1 does not appear to have an equivalent function as Six2 in inducing differentiation, because Six1 binding to the Six2 target sites at the Fgf8, Pax8 or Wnt4 was not detected. Our observation that the rapid commitment to an RV fate occurring in *Six2* ^*−/−*^ did not happen in *Eya1* ^*Six1/+*^ *;Six2* ^*−/−*^ suggests that Six1 inhibits premature nephron differentiation in the absence of Six2. We thus speculate that the nuclear Eya1 in *Eya1* ^*Six1/+*^ *;Six2* ^*−/−*^ may play a role in countering nephron progenitor cell differentiation. Alternatively, Six1 may indirectly inhibit differentiation through the regulation of other factors. In supporting this, Six1 enrichment was detected at the Ezh1 and Ezh2 promoter regions that are associated with H3K27ac-deposition and co-occupied by Six2 (Figure 7). Since previous studies have found that inactivation of both Ezh1/Ezh2 leads to unscheduled activation of Wnt4 (Liu et al., 2020), Six1 may regulate the expression of these two genes to prevent ectopic Wnt4 activation. These two possibilities could in conjunction explain the requirement of Six1 for preventing rapid epithelialization of the progenitors in *Eya1* ^*Six1/+*^ *;Six2* ^*−/−*^. This also suggests that Six1 is able to form transcriptional complexes capable of associating with the same regulatory DNA elements co-occupied by Six2 at the Ezh1 and Ezh2. Thus, our finding provides insights into why nephron progenitors undergo rapid RV formation in the absence of Six2.

In summary, our results indicate that the Six1 and Six2 genes of the *Drosophila so* subfamily have diverged in function by the acquisition of different regulatory patterns and special biochemical properties. Previous studies have focused on the conserved SD and HD in recognizing subgroup specific sites and common targets. In human fetal kidneys, SIX1 and SIX2 cross-regulate each other (O'Brien et al., 2016), which is thought to be critical to maintain the absolute levels of SIX proteins that are necessary for generating approximately seventy times more nephrons in a human kidney than a mouse kidney. However, SIX2 apparently has a more prominent roles than SIX1 and ∼79.2% of SIX2 target sites are not targeted by SIX1. As SIX1 and SIX2 physically interact when coexpressed in HEK293 cells (O'Brien et al., 2016), it is possible that small percentage of SIX1 and SIX2 may form complexes that may be below the detection threshold by co-immunoprecipitation. Nonetheless, our evidence for the functional difference between the mouse Six1 and Six2 suggests that the evolutionary division of these two genes has occurred at the levels of both protein function and their gene-expression programs, with particular cell types manifesting greater dependence on the expression of one or the other or both. Future research to explore whether the divergent C-terminal domains influence the molecular and biochemical rules governing the specificity of these proteins may help us understand how the mechanisms that control spatial aspects of nephrogenesis have been modified during evolution. Lastly, it is worth mentioning that a recent study explored the potential of SIX1 with EYA1 and SNAI2 in directly reprogramming HK2 cells into nephron progenitor-like cells (Vanslambrouck et al., 2019). Our finding that Six1 and Six2 have non-equivalent biochemical properties provides valuable information for the development of nephron reprogramming strategies.

## Data Availability

The datasets presented in this study can be found in online repositories. The names of the repository/repositories and accession number(s) can be found below: NCBI with GSE ID is GSE189131 (https://www.ncbi.nlm.nih.gov/geo/query/acc.cgi?acc=GSE189131).
